# Endo-lysosomal TRP mucolipin-1 channels trigger global ER Ca^2+^ release and Ca^2+^ influx

**DOI:** 10.1242/jcs.190322

**Published:** 2016-10-15

**Authors:** Bethan S. Kilpatrick, Elizabeth Yates, Christian Grimm, Anthony H. Schapira, Sandip Patel

**Affiliations:** 1 Department of Cell and Developmental Biology, University College London, Gower Street, London WC1E 6BT, UK; 2 Center for Integrated Protein Science CIPSM and Department of Pharmacy – Center for Drug Research, Ludwig-Maximilians-Universität München, München 81377, Germany; 3 Department of Clinical Neurosciences, Institute of Neurology, University College London, London NW3 2PF, UK

**Keywords:** Ca^2+^, Lysosomes, TRP channels, Endoplasmic reticulum

## Abstract

Transient receptor potential (TRP) mucolipins (TRPMLs), encoded by the MCOLN genes, are patho-physiologically relevant endo-lysosomal ion channels crucial for membrane trafficking. Several lines of evidence suggest that TRPMLs mediate localised Ca^2+^ release but their role in Ca^2+^ signalling is not clear. Here, we show that activation of endogenous and recombinant TRPMLs with synthetic agonists evoked global Ca^2+^ signals in human cells. These signals were blocked by a dominant-negative TRPML1 construct and a TRPML antagonist. We further show that, despite a predominant lysosomal localisation, TRPML1 supports both Ca^2+^ release and Ca^2+^ entry. Ca^2+^ release required lysosomal and ER Ca^2+^ stores suggesting that TRPMLs, like other endo-lysosomal Ca^2+^ channels, are capable of ‘chatter’ with ER Ca^2+^ channels. Our data identify new modalities for TRPML1 action.

## Abstract (precis)

**Summary:** The endolysosomal ion channel TRP mucolipin 1 was thought to mediate local Ca^2+^ signals. However, as reported here, it can also mediate global elevations in Ca^2+^.

## INTRODUCTION

The remarkable versatility of Ca^2+^ as an intracellular messenger is underpinned by the spatiotemporal organisation of Ca^2+^ signals (Berridge et al., 2003). Targeted delivery of Ca^2+^ to a particular subcellular locale is one way in which Ca^2+^ can selectively influence a particular cell outcome (e.g. secretion). Equally, global Ca^2+^ signals, which are often oscillatory, can also be selective through information encoded in oscillation frequency. Interplay between Ca^2+^ release from intracellular Ca^2+^ stores and Ca^2+^ influx from the extracellular space ensures tight control of Ca^2+^ levels and their downstream targets (Clapham, 2007). Although we have gained much mechanistic insight into the mobilisation of ER Ca^2+^ stores and the subsequent entry of Ca^2+^ across the plasma membrane that ensues (Prakriya and Lewis, 2015), we know relatively little about Ca^2+^ handling by acidic Ca^2+^ stores such as lysosomes (Patel and Muallem, 2011).

Lysosomes are best known for their degradative role but they maintain an intraluminal Ca^2+^ concentration (∼500 µM) similar to the ER (Christensen et al., 2002; Lloyd-Evans et al., 2008). They are likely filled by Ca^2+^-H^+^ exchange (Melchionda et al., 2016) and express members of the transient receptor potential mucolipin (TRPML) and two-pore channel (TPC) families to effect Ca^2+^ release (Grimm et al., 2012; Kiselyov et al., 2012; Patel, 2015; Waller-Evans and Lloyd-Evans, 2015). Three TRPML isoforms are present in humans (García-Añoveros and Wiwatpanit, 2014). TRPML1 (encoded by *MCOLN1*) is ubiquitously expressed and targets to lysosomes through di-leucine motifs (Vergarajauregui and Puertollano, 2006). It is activated by the endo-lysosomal phosphoinositide phosphatidylinositol 3,5-bisphosphate [PI(3,5)P_2_] (Dong et al., 2010) and is a non-selective cation channel permeable to a number of ions including Ca^2+^ and Fe^2+^ (Dong et al., 2008). Importantly, mutation of TRPML1 results in the lysosomal storage disorder mucolipidosis type IV (Bargal et al., 2000). This neurodegenerative disorder is characterised by build-up of various lipid species in lysosomes, suggesting a role for TRPML1 in lipid trafficking. It has long been recognised that localised Ca^2+^ release from the endo-lysosomal system can influence vesicular fusion and fission events (Pryor et al., 2000). Specifically, Ca^2+^ release through TRPMLs likely underlies functional roles in vesicular trafficking as well as lysosomal exocytosis (Samie et al., 2013), autophagy (Medina et al., 2015) and regulated secretion (Park et al., 2016). Notably, recently described TRPML agonists have proven beneficial in correcting trafficking defects not only in mucolipidosis type IV (Chen et al., 2014) but also in Niemann–Pick type C disease (Shen et al., 2012) and HIV-associated dementia (Bae et al., 2014). Defective TRPML activity has also been implicated in Alzheimer's disease (Lee et al., 2015). Despite their demonstrable (patho)physiological relevance, TRPML-mediated Ca^2+^ signals are difficult to resolve due to their localised nature. To overcome this, a number of recent studies have used genetically encoded Ca^2+^ indicators such as GCaMP-3 or GECO fused directly to TRPML1 to record Ca^2+^ signals in response to agonist activation (Bae et al., 2014; Cao et al., 2015; Medina et al., 2015; Shen et al., 2012). These signals are suggested to reflect local peri-lysosomal Ca^2+^ release. But it is not clear how the relatively high-affinity Ca^2+^ indicators used distinguish between local and global Ca^2+^ signals.

In addition to supporting local Ca^2+^ fluxes required for endocytic ‘well-being’ (Hockey et al., 2015), lysosomes can also initiate global Ca^2+^ signals during signalling. This process has been established in the context of the actions of the messenger nicotinic acid adenine dinucleotide phosphate (NAADP) (Galione, 2015). According to the trigger hypothesis, increases in NAADP levels activate endo-lysosomal Ca^2+^ channels, generating local Ca^2+^ signals that sensitise neighbouring Ca^2+^ release channels on the ER. Activation of the latter (on the more substantial store) results in global Ca^2+^ release. Indeed, in fibroblasts, direct mobilisation of lysosomal Ca^2+^ stores through osmotic permeabilisation is sufficient to trigger ER-dependent complex Ca^2+^ signals (Kilpatrick et al., 2013). Physiologically, it is the TPCs that have emerged as the target channels for NAADP (Brailoiu et al., 2009; Calcraft et al., 2009; Patel, 2015) although a role for TRPMLs has also been advanced (Zhang et al., 2011, but see Yamaguchi et al., 2011). Coupling between TPCs and ER Ca^2+^ channels likely occurs at membrane contact sites between lysosomes and the ER (Kilpatrick et al., 2013; Patel and Brailoiu, 2012; Penny et al., 2014). Although TRPMLs can evoke global Ca^2+^ entry, at least when mutated as exemplified by the Va mutation in TRPML3 (encoded by *MCOLN3*) (Grimm et al., 2007; Kim et al., 2007), whether they support lysosome–ER cross talk is not known.

Here, we take advantage of TRPML agonists ML-SA1 (Shen et al., 2012) and MK6-83 (Chen et al., 2014) to probe the Ca^2+^ permeability of TRPMLs in an intact cell setting. We show that activation of TRPML1 unexpectedly evokes global Ca^2+^ signals. Mechanistically, we dissect these signals into release and influx components implicating both ER and extracellular Ca^2+^, in addition to lysosomal Ca^2+^, for their genesis. Our data suggest that the action of TRPML1 is not limited to local lysosomal Ca^2+^ signalling.

## RESULTS

### TRPML agonists evoke global Ca^2+^ signals

**Fig. 1.**
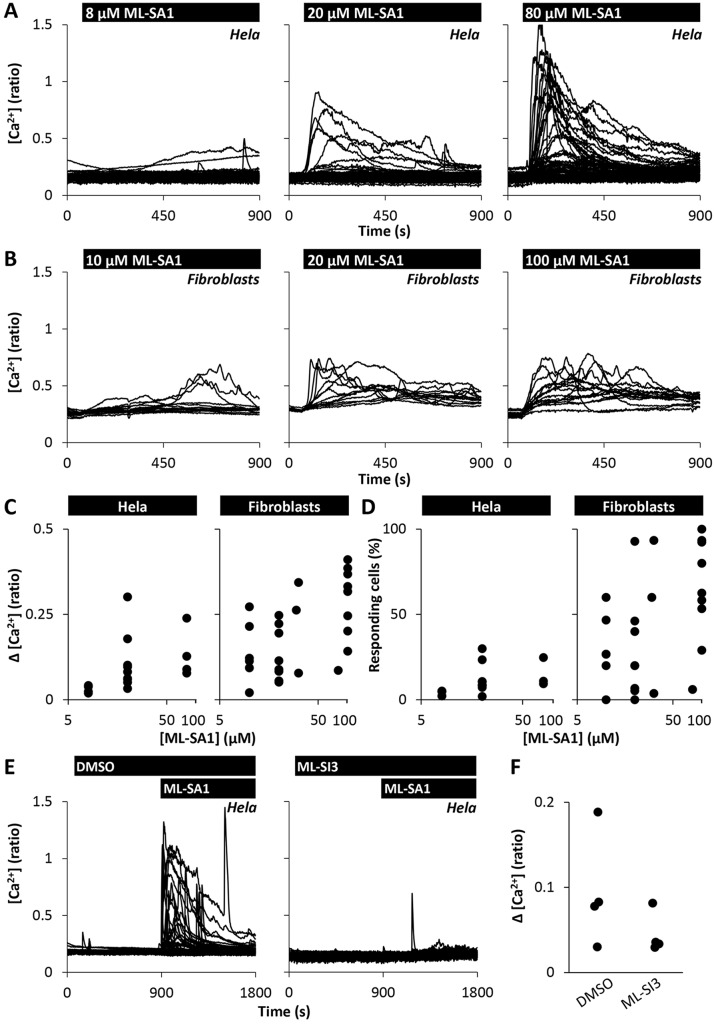
**The TRP mucolipin agonist ML-SA1 evokes global Ca^2+^ signals.** (A–D) Effect of ML-SA1 on cytosolic Ca^2+^ levels. Cytosolic Ca^2+^ levels of individual Fura-2-loaded HeLa cells (A) or fibroblasts (B) from an exemplar population stimulated with increasing concentrations of ML-SA1. (C,D) Summary data quantifying the peak response (C) and percentage of responsive cells within the population (D). Each data point represents an independent repetition. A total of 350–788 HeLa cells and 81–140 fibroblasts were analysed. (E,F) Effect of the mucolipin inhibitor ML-SI3 on agonist-evoked Ca^2+^ signals. (E) Cytosolic Ca^2+^ levels of HeLa cells stimulated with 20 µM ML-SA1 in the presence of vehicle (0.1% DMSO) or 10 µM ML-SI3. (F) Summary data for the experiments in E (366–406 cells).

Fusion proteins comprising TRPML1 and either GCaMP-3 (Shen et al., 2012) or GECO (Cao et al., 2015) have been used to resolve presumed local Ca^2+^ fluxes from lysosomes upon TRPML1 activation. But as shown in Fig. 1A and Movie 1, Ca^2+^ responses to ML-SA1 (20 µM) were readily resolvable in a proportion of Hela cells loaded with the fluorescent Ca^2+^ indicator Fura-2. This organic dye distributes throughout the cytosol and thus records bulk changes in cytosolic Ca^2+^. ML-SA1 responses were concentration dependent (Fig. 1A). Similar results were obtained using primary cultured human fibroblasts. As in HeLa cells, ML-SA1 evoked strong Ca^2+^ responses in Fura-2-loaded cells in a concentration-dependent manner (Fig. 1B; Movie 2). The amplitude of the Ca^2+^ signals and the proportion of responsive cells are quantified in Fig. 1C,D. Fibroblasts were more responsive than HeLa cells, although we noted considerable variability between experiments, particularly for fibroblasts (Fig. 1D). To probe specificity, we examined the effects of the new TRPML antagonists ML-SI1 and ML-SI3 (Samie et al., 2013). ML-SI1 could not be used because it evoked Ca^2+^ signals (Fig. S1A). ML-SI3, however, inhibited ML-SA1-evoked Ca^2+^ signals (Fig. 1E,F). Collectively, these data suggest that, contrary to the prevailing view, activation of endogenous TRPMLs is capable of evoking global Ca^2+^ signals.

### Agonist-evoked Ca^2+^ signals require TRPML1

**Fig. 2.**
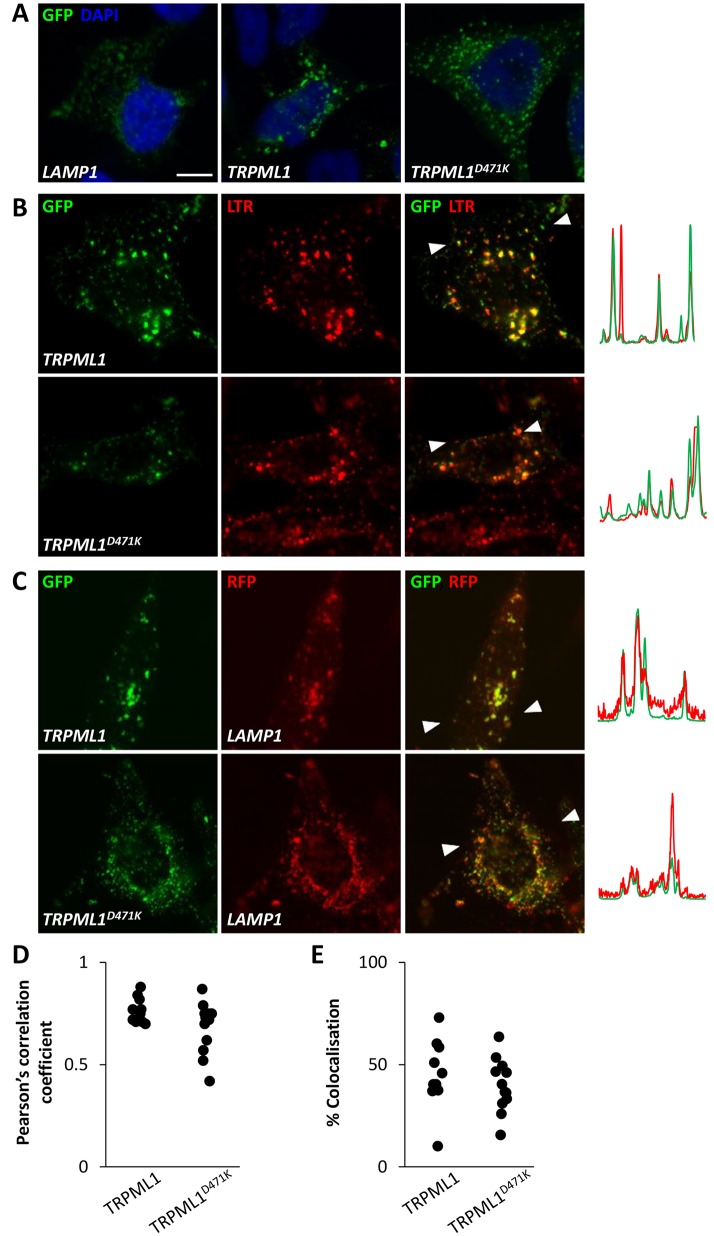
**Subcellular distribution of TRPML1.** (A) Confocal fluorescence images (green) of fixed HeLa cells expressing GFP-tagged LAMP1 (left), TRPML1 (middle) or TRPML1^D471K^ (right). Nuclei were stained using DAPI (blue). (B,C) Confocal fluorescence images of cells expressing GFP–TRPML1 or GFP–TRPML1^D471K^ and either colabelled with Lysotracker^®^ Red (LTR) and imaged live (B) or co-transfected with LAMP1-mRFP and imaged following fixation (C). Overlays of images are shown in the right panels where arrows delineate the regions from which red–green intensity plots were derived. Scale bar: 10 µm. (D,E) Summary data of experiments in C quantifying Pearson's correlation coefficients (D) and the percentage of vesicles showing colocalisation (E). Each point represents an individual cell from two independent transfections.

To investigate the mechanism underlying agonist-evoked Ca^2+^ signals, we examined the effects of expressing active TRPML1 and inactive TRPML1 mutated within the pore region (TRPML1^D471K^) (Yamaguchi et al., 2011). Confocal analyses of HeLa cells expressing TRPML1 and TRPML1^D471K^ revealed a comparable punctate distribution consistent with localisation to lysosomes (Fig. 2A). Accordingly, there was marked colocalisation of both proteins with lysotracker (a fluorescent acidotrope) and LAMP1 (a lysosomal marker) (Fig. 2B–E).

**Fig. 3.**
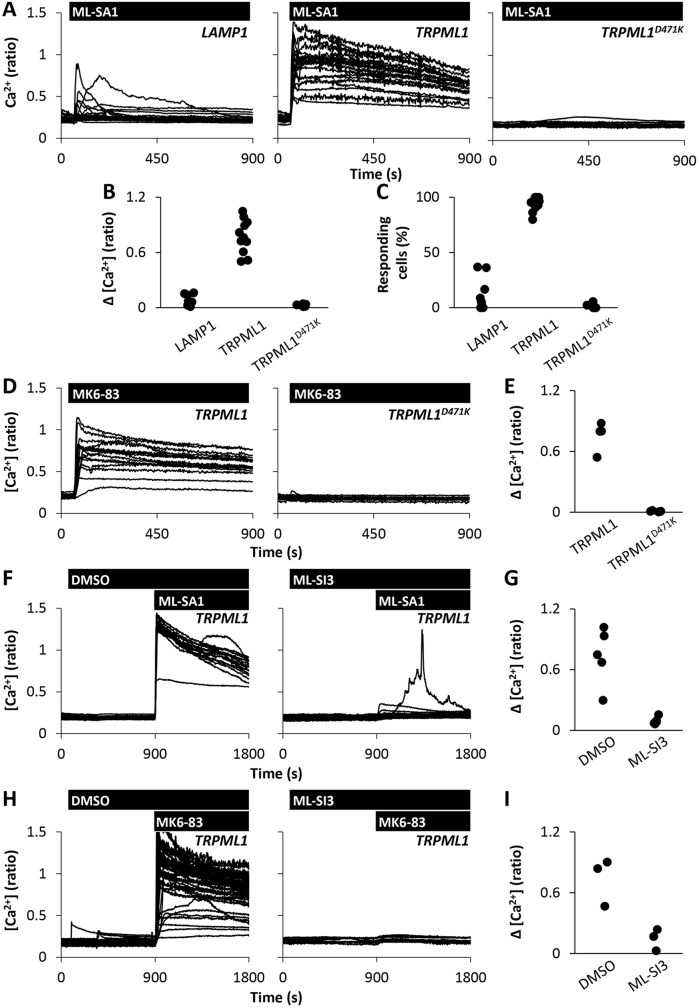
**Agonist-evoked Ca^2+^ signals require TRPML1.** (A–C) Effect of TRPML expression on ML-SA1-evoked Ca^2+^ signals. (A) Cytosolic Ca^2+^ levels of individual LAMP1, TRPML1- or TRPML1^D471K^-expressing HeLa cells stimulated with 20 µM ML-SA1. (B,C) Summary data of experiments in A where each data point represents an independent repetition. The total number of cells analysed was 193–367. (D,E) Effect of TRPML expression on MK6-83-evoked Ca^2+^ signals. (D) Cytosolic Ca^2+^ levels of individual TRPML1- or TRPML1^D471K^-expressing HeLa cells stimulated with 20 µM MK6-83. (E) Summary data of experiments in D (111–128 cells). (F–I) Effect of ML-SI3 on agonist-evoked Ca^2+^ signals. Cytosolic Ca^2+^ levels of individual TRPML1-expressing HeLa cells stimulated with 20 µM ML-SA1 (F) or 20 µM MK6-83 (H) in the presence of vehicle (0.1% DMSO) or 10 µM ML-SI3. Summary data are shown in panels (G; 120 cells) and (I; 101–107 cells), respectively.

In control experiments, ML-SA1 evoked Ca^2+^ signals in cells expressing LAMP1 (Fig. 3A). The responses were similar to those in un-transfected cells (Fig. 1). In cells expressing TRPML1, however, there was a large potentiation of the Ca^2+^ signal (Fig. 3A) such that essentially all of the cells responded (Fig. 3B,C). The potentiating effect of TRPML1 was specific because it was not observed with TRPML1^D471K^ (Fig. 3A–C). Similar results were obtained in fibroblasts (Fig. S1C,D) although this analysis was more limited due to the difficulty in transfecting these cells. Notably, ML-SA1-evoked Ca^2+^ signals were inhibited in cells expressing TRPML1^D471K^ relative to cells expressing LAMP1 (Fig. 3A–C). The inhibitory effect of TRPML1^D471K^ is likely due to dominant-negative activity resulting from oligomerisation with endogenous TRPML1 (Venkatachalam et al., 2006).

We also tested the effect of the structurally distinct mucolipin analogue MK6-83. Like ML-SA1, MK6-83 evoked large Ca^2+^ signals in cells expressing TRPML1 but not TRPML1^D471K^ (Fig. 3D,E). Ca^2+^ signals evoked by both ML-SA1 and MK6-83 in TRPML1-expressing cells were blocked by ML-SI3 (Fig. 3F–I). These molecular and chemical analyses establish the requirement for TRPML1 in global Ca^2+^ signalling.

### TRPML1 couples lysosomal and ER Ca^2+^ release

**Fig. 4.**
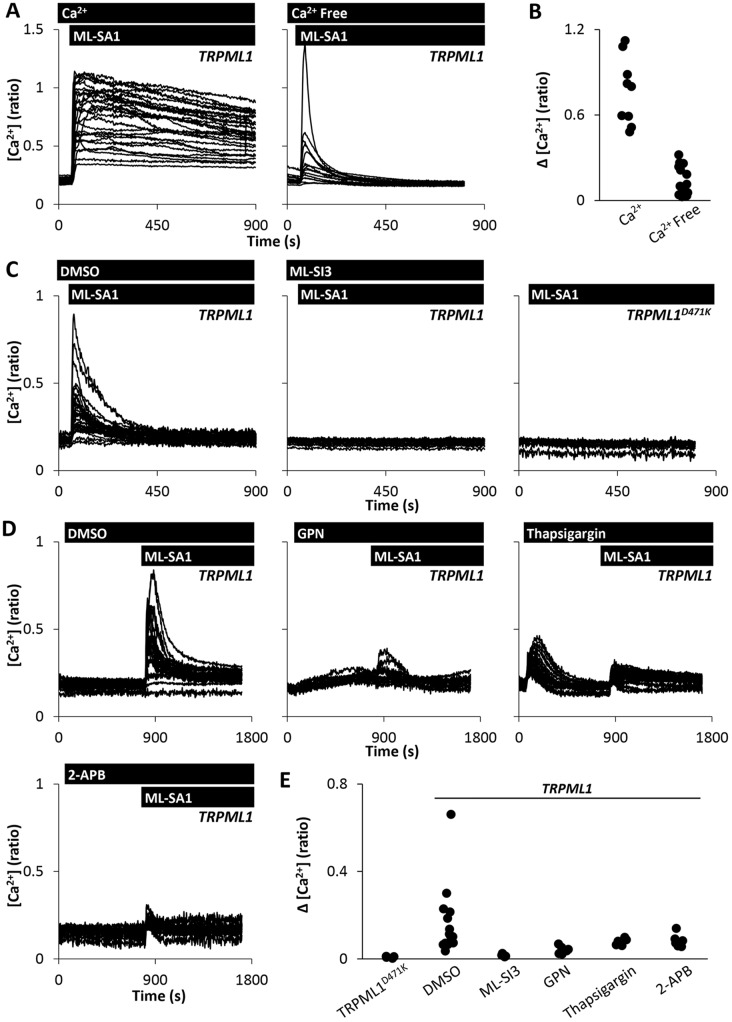
**TRPML1 couples lysosomal and ER Ca^2+^ release.** (A,B) Effect of extracellular Ca^2+^ on agonist-evoked Ca^2+^ responses. (A) Cytosolic Ca^2+^ levels of TRPML1-expressing HeLa cells stimulated with 20 µM ML-SA1 in Ca^2+^-containing or Ca^2+^-free Hepes-buffered saline. (B) Summary data of experiments in A where each data point represents an independent repetition. The total number of cells analysed was 184–284. (C) Cytosolic Ca^2+^ levels of TRPML1 or TRPML1^D471K^-expressing HeLa cells stimulated with 20 µM ML-SA1 with or without 10 µM ML-SI3 in Ca^2+^-free medium. (D,E) Effect of disrupting Ca^2+^ stores on agonist-evoked Ca^2+^ responses. (D) Cytosolic Ca^2+^ levels of TRPML1-expressing HeLa cells stimulated with 20 µM ML-SA1 following pre-treatment with DMSO, 200 µM GPN, 1 µM thapsigargin or 100 µM 2-APB. (E) Summary data of experiments in D (71–462 cells). Experiments were performed in Ca^2+^-free medium.

To define the Ca^2+^ sources underlying TRPML1-dependent Ca^2+^ signals, we first established the relative contribution of store release and Ca^2+^ entry to the evoked responses. As shown in Fig. 4A,B, removal of extracellular Ca^2+^ reduced the peak response upon ML-SA1 stimulation and eliminated the sustained phase. ML-SA1-evoked Ca^2+^ signals in Ca^2+^-free medium were TRPML1 dependent, as judged by the lack of ML-SA1-evoked Ca^2+^ signals in TRPML1^D471K^-expressing cells and upon treatment with ML-SI3 (Fig. 4C). These data suggest that ML-SA1 evokes both Ca^2+^ release and Ca^2+^ influx.

We used the lysosomotropic agent Gly-Phe β-naphthylamide (GPN) to disrupt lysosomes. This dipeptide causes osmotic destabilisation of lysosomes and other cathepsin-C-positive compartments (Jadot et al., 1984). GPN treatment resulted in a rapid loss of Lysotracker^®^ red fluorescence consistent with its action on lysosomes (Fig. S1E,F) and inhibited the responses to ML-SA1 indicating a requirement for lysosomes in ML-SA1-evoked Ca^2+^ release (Fig. 4D). These data are consistent with the lysosomal localisation of TRPMLs (Fig. 2). However, despite the complete compromise of lysosomes upon GPN treatment, the resulting Ca^2+^ signals were modest relative to those evoked by ML-SA1 (Fig. 4D). This suggested that ML-SA1 did not exclusively release Ca^2+^ from the lysosomes.

We considered the possibility that activation of TRPMLs might be followed by Ca^2+^ release from the ER in a manner similar to activation of TPCs by NAADP (Cancela et al., 1999; Patel et al., 2010). To test this, we examined the effects of ML-SA1 after depleting ER Ca^2+^ stores with thapsigargin. As shown in Fig. 4D, thapsigargin treatment blocked the responses to ML-SA1 indicating a clear requirement for ER Ca^2+^ stores in ML-SA1 action. To further probe the role of the ER, we used 2-APB to block inositol 1,4,5-trisphosphate (IP_3_) receptors. As shown in Fig. 4D, 2-APB (100 µM) inhibited ML-SA1-evoked responses. A summary of these data is provided in Fig. 4E. We conclude that activation of TRPMLs on lysosomes triggers Ca^2+^ release from the ER.

### TRPML1 mediates Ca^2+^ influx

**Fig. 5.**
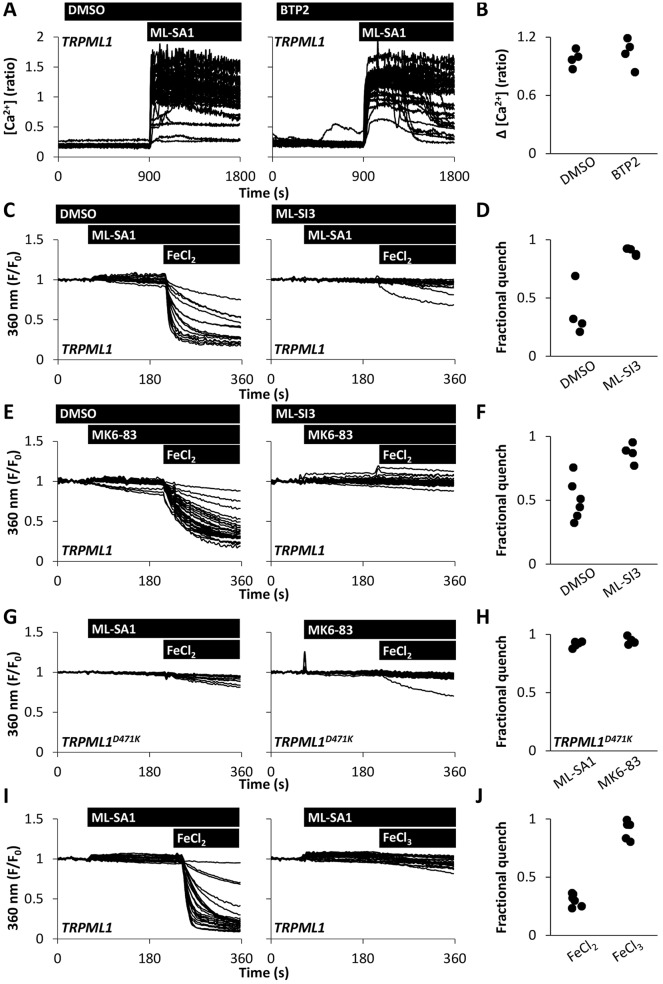
**TRPML1 mediates Ca^2+^ influx.** (A,B) Effect of blocking store-operated Ca^2+^ influx on agonist-evoked Ca^2+^ responses. (A) Cytosolic Ca^2+^ levels of TRPML1-expressing HeLa cells stimulated with 20 µM ML-SA1 in the presence of vehicle (0.1% DMSO) or 20 µM BTP2. (B) Summary data of experiments in A where each data point represents an independent repetition. The total number of cells analysed was 151–173. (C–J) Effect of TRPML1 activation on Fe ion entry. HeLa cells expressing TRPML1 were stimulated with 20 µM ML-SA1 (C) or MK6-83 (E) and then 1 mM FeCl_2_ in the presence of vehicle (0.1% DMSO) or 10 µM ML-SI3. HeLa cells expressing TRPML1^D471K^ were stimulated with either 20 µM ML-SA1 or MK6-83 (G). HeLa cells expressing TRPML1 were stimulated with 20 µM ML-SA1 and either 1 mM FeCl_2_ or 1 mM FeCl_3_ (I). Experiments were performed in nominally Ca^2+^-free medium. Fluorescence signals (denoted F) were recorded after excitation at the isosbestic wavelength of Fura-2 (360 nm) and normalised to that at the beginning of recording (denoted F_0_). Summary data (D,F,H,J; for C,E,G and I, respectively) quantifying the fractional loss of fluorescence 144s after addition of Fe^2+^ or Fe^3+^ under the various conditions (80–169 cells).

Finally, we examined the nature of the Ca^2+^ entry pathway evoked by TRPML1 activation. Because store-operated Ca^2+^ entry is a ubiquitous pathway underlying Ca^2+^ influx in non-excitable cells, we tested the effects of the Ca^2+^ entry inhibitor BTP2. BTP2 (20 µM) completely prevented Ca^2+^ signals evoked by Ca^2+^ add-back following depletion of ER Ca^2+^ stores with thapsigargin (Fig. S2A,B). However, BTP2 did not affect the amplitude of Ca^2+^ signals evoked by ML-SA1 (Fig. 5A,B) although it did slow the rate of rise (Fig. S2C). These data suggest that store-operated Ca^2+^ entry does not play a major role in Ca^2+^ signals evoked by TRPML1 activation.

The above data raised the possibility that TRPML1 might mediate Ca^2+^ entry more directly. To test this, we examined the effects of ML-SA1 on Fe^2+^ entry. Fe^2+^ permeates TRPML1 and, like several other metal ions, quenches Fura-2 fluorescence (Dong et al., 2008). Fura-2-loaded HeLa cells were therefore challenged with FeCl_2_ (1 mM) in nominally Ca^2+^-free medium after stimulation with ML-SA1. FeCl_2_ induced a significant quench of Fura-2 fluorescence in cells expressing TRPML1 (Fig. 5C). This effect was blocked by ML-SI3 (Fig. 5C,D) and was not observed in cells expressing TRPML1^D471K^ (Fig. 5G,H). Similar results were obtained upon stimulation with MK6-83 (Fig. 5E–H). Challenge with FeCl_3_ (1 mM) after stimulation with ML-SA1 was without effect (Fig. 5I,J) attesting to specificity. BTP2 slowed Fe^2+^ entry (Fig. S2D,E), however, add-back of Fe^2+^ following depletion of Ca^2+^ stores with thapsigargin did not result in a significant quench (Fig. S2F,G).

Collectively, these data provide evidence that TRPML1 supports divalent cation entry across the plasma membrane.

## DISCUSSION

In this study, we have shown that activation of TRPMLs by two structurally distinct agonists, in both a human cell line and primary cultures, evokes global Ca^2+^ signals. These data indicate that TRPMLs do not solely mediate local Ca^2+^ release events from endo-lysosomal compartments. Indeed, we provide evidence that activation of TRPMLs can evoke both Ca^2+^ release and Ca^2+^ entry.

We have dissected global Ca^2+^ release signals into both lysosomal and ER components. There is much evidence supporting the transmission of Ca^2+^ signals from endo-lysosomes to the ER but to date such ‘chatter’ has been ascribed exclusively to the actions of NAADP and TPCs (Patel and Brailoiu, 2012). Our study extends such coupling to TRPMLs, raising the possibility that other Ca^2+^-permeable endo-lysosomal channels such as P2X4 receptors and TRPM2 might also functionally couple to ER Ca^2+^ release channels (Patel and Cai, 2015).

Inhibition of TRPML1 by plasma membrane PI(4,5)P_2_ is thought to ensure channel activation only in endo-lysosomal compartments (Zhang et al., 2012). In accordance with this idea, basal (unstimulated) whole-cell currents are not detectable in TRPML1-expressing cells. SF-51 (Grimm et al., 2010), the precursor to ML-SA1, however, has been reported to mediate a TRPML1 plasma membrane current (Zhang et al., 2012) consistent with our Ca^2+^ and Fe^2+^ entry measurements. Divalent cation entry might therefore result from targeting of a small fraction of TRPML1 to the plasma membrane. Although blockade of store-operated channels reduced TRPML1-dependent Ca^2+^ and Fe^2+^ entry (albeit modestly), the amplitude of the Ca^2+^ signal upon maximal Ca^2+^ ER store depletion was substantially smaller than that evoked by agonist activation. Indeed, store-operated Fe^2+^ entry was not demonstrable under our conditions. These data raise the possibility that BTP2 might directly inhibit TRPML1 similar to its effects on TRP-canonical (TRPC) channels (He et al., 2005).

The multi-compartment actions of TRPML1 offer fresh perspective on interpreting Ca^2+^ signals recorded using Ca^2+^ reporters fused to TRPML1 (Bae et al., 2014; Cao et al., 2015; Medina et al., 2015; Shen et al., 2012). We suggest that such signals might additionally reflect Ca^2+^ release from the ER and Ca^2+^ entry. Indeed, defects in Ca^2+^ signals reported using such indicators in scenarios of TRPML1 dysfunction are perplexing given that expression of TRPML1 is able to rescue phenotypic defects such as lysosomal storage (Cao et al., 2015; Shen et al., 2012). Use of inert lysosomal proteins for targeting (McCue et al., 2013) and/or low-affinity Ca^2+^ indicators, to better insulate against bulk cytosolic Ca^2+^ changes, might aid in isolating local lysosomal Ca^2+^ release events. Finally, how TRPMLs are endogenously activated remains to be established, but PI(3,5)P_2_ is a clear candidate raising the possibility that changes in its levels in response to cues such as growth factors might regulate global Ca^2+^ dynamics.

## MATERIALS AND METHODS

### Cell culture

HeLa cells and primary cultured human skin fibroblasts from healthy individuals (Kilpatrick et al., 2013) were maintained in Dulbecco's modified Eagle's medium (DMEM) supplemented with 10% (v/v) fetal bovine serum, 100 units/ml penicillin and 100 µg/ml streptomycin (all from Invitrogen) at 37°C in a humidified atmosphere with 5% CO_2_. Cells were passaged using trypsin (HeLa) or scraping (fibroblasts) and plated onto 13-mm glass coverslips prior to microscopy. For HeLa cells, coverslips were coated with poly-L-lysine (Sigma). Cells were transfected with plasmids using Lipofectamine 2000 according to the manufacturer's instructions. Plasmids encoding the following proteins were described previously: C-terminally GFP-tagged LAMP1 (Falcón-Pérez et al., 2005), C-terminally mRFP-tagged LAMP1 (Sherer et al., 2003) and N-terminally GFP-tagged wild-type or D471K human TRPML1 (Yamaguchi et al., 2011).

### Cell labelling

All live imaging experiments were performed in HEPES-buffered saline (HBS) comprising (in mM): 1.25 KH_2_PO_4_, 2 CaCl_2_, 2 MgSO_4_, 3 KCl, 156 NaCl, 10 glucose, and 10 HEPES (pH 7.4; all from Sigma). For measurement of cytosolic Ca^2+^ concentration and Fe^2+^ or Fe^3+^ entry, cells were incubated with Fura-2 AM (2.5 µM) and 0.005% v/v pluronic acid (from Invitrogen) for 1 h. For measurement of lysosome distribution, cells were incubated with Lysotracker^®^ Red (100 nM) (Invitrogen) for 15 min. After labelling, cells were washed with HBS and mounted in a 1-ml imaging chamber (Biosciences Tools) prior to microscopy. For measurement of colocalisation, cells were co-transfected with GFP–TRPML1 or GFP–TRPML1^D471K^ together with LAMP1–mRFP and fixed with 4% paraformaldehyde prior to confocal microscopy. For visualisation of nuclei, cells were incubated with 1 µg/ml DAPI for 5 min.

### Epifluorescence microscopy

Epifluorescence images were captured every 3 s with a cooled coupled device camera (TILL photonics) attached to an Olympus IX71 inverted fluorescence microscope fitted with a 20× objective, and a monochromator light source. Fura-2 was excited at 340, 360 or 380 nm, and Lysotracker^®^ Red at 560 nm. Emitted fluorescence was captured using a 440-nm long-pass filter (Fura-2) and 590-nm filter (Lysotracker^®^ Red). TRPML1-expressing cells were identified by monitoring fluorescence of GFP (excitation 488 nm, emission 505 nm). Cells were stimulated with ML-SA1, GW405833 hydrochloride (Sigma; also known as ML-SI1, Haoxing Xu, Department of Molecular, Cellular, and Developmental Biology, University of Michigan, USA, personal communication), ML-SI3 (a kind gift from Haoxing Xu), MK6-83 synthesised as described previously (Chen et al., 2014), GPN (Santa Cruz Biotechnology), BTP2 (Sigma), thapsigargin (Merck), 2-aminoethoxydiphenyl borate (2-APB, Sigma). Where indicated, Ca^2+^ was omitted from the HBS (nominally Ca^2+^-free) or replaced with 1 mM EGTA (Ca^2+^-free). For Fe^2+^ or Fe^3+^ quench experiments, cells were stimulated using freshly prepared 100 mM stock solutions of either FeCl_2_ or FeCl_3_ (Sigma). The maximal fluorescence ratio change (Ca^2+^) or the fractional quench at 360 nm excitation (Fe^2+^ or Fe^3+^) were quantified on an individual cell basis and averaged for all cells in a given field of view. Experiments were repeated at least three times and all individual data points plotted. The total number of cells analysed is stated in the legends.

### Confocal microscopy

Confocal images were captured using an LSM510 confocal scanner (Zeiss) attached to a Zeiss Axiovert 200M inverted microscope fitted with a 63× Plan Apochromat water-immersion objective. DAPI, GFP and Lysotracker^®^ Red or mRFP were excited at 364 nm, 488 nm and 543 nm, and emitted fluorescence was captured using 385–470 nm, 505–550 nm and 560–615 nm band-pass filters, respectively. Pearson's correlation coefficients were calculated from *z*-stacks (1-µm intervals, 8–12 slices) using the ImageJ plugin Coloc2. The fraction of colocalised vesicles was calculated from the middle slice or slices using the ImageJ plugin SQUASSH.
